# Release of Tensile Strain on Engineered Human Tendon Tissue Disturbs Cell Adhesions, Changes Matrix Architecture, and Induces an Inflammatory Phenotype

**DOI:** 10.1371/journal.pone.0086078

**Published:** 2014-01-21

**Authors:** Monika L. Bayer, Peter Schjerling, Andreas Herchenhan, Cedric Zeltz, Katja M. Heinemeier, Lise Christensen, Michael Krogsgaard, Donald Gullberg, Michael Kjaer

**Affiliations:** 1 Institute of Sports Medicine, Department of Orthopedic Surgery M, Bispebjerg Hospital and Center for Healthy Aging, Faculty of Health and Medical Sciences, University of Copenhagen, Copenhagen, Denmark; 2 Department of Biomedicine, University of Bergen, Bergen, Norway; 3 Department of Pathology, Bispebjerg Hospital, University of Copenhagen, Copenhagen, Denmark; 4 Section for Sports Traumatology, Department of Orthopedic Surgery M, Bispebjerg Hospital, University of Copenhagen, Copenhagen, Denmark; Université de Technologie de Compiègne, France

## Abstract

Mechanical loading of tendon cells results in an upregulation of mechanotransduction signaling pathways, cell-matrix adhesion and collagen synthesis, but whether unloading removes these responses is unclear. We investigated the response to tension release, with regard to matrix proteins, pro-inflammatory mediators and tendon phenotypic specific molecules, in an *in vitro* model where tendon-like tissue was engineered from human tendon cells. Tissue sampling was performed 1, 2, 4 and 6 days after surgical de-tensioning of the tendon construct. When tensile stimulus was removed, integrin type collagen receptors showed a contrasting response with a clear drop in integrin subunit α_11_ mRNA and protein expression, and an increase in α_2_ integrin mRNA and protein levels. Further, specific markers for tendon cell differentiation declined and normal tendon architecture was disturbed, whereas pro-inflammatory molecules were upregulated. Stimulation with the cytokine TGF-β1 had distinct effects on some tendon-related genes in both tensioned and de-tensioned tissue. These findings indicate an important role of mechanical loading for cellular and matrix responses in tendon, including that loss of tension leads to a decrease in phenotypical markers for tendon, while expression of pro-inflammatory mediators is induced.

## Introduction

Tendon is a collagen-rich tissue that plays an indispensable role in locomotion and postural control, and its parallel arrangement of collagen fibrils along the tensional axis allows tendons to withstand high forces [1]. The cells within the tendon are organized in a parallel alignment located in between the collagen fibrils with elongated cell nuclei and long cellular protrusions [2], [3]. Tensile strain is a major mechanical stimulus that tendons are subjected to, and to which tendons can adapt [4], and adaptive advantageous responses to tensile loading have even been demonstrated in healing tendon tissue [5]. Tendon injuries are a frequent problem and in general, the regeneration process of tendon pathologies is poor and often leads to fibrotic changes and inferior function of the tissue [6], [7], [8], [9], [10]. It has remained largely unknown what role the mechanical environment plays in the activation of catabolic changes of human tendon cells, which might explain the development of pathological changes and complication during the regeneration process. During embryonic development, tendon progenitor cells express a certain set of genes, which are associated with different stages of tendon formation [11], [12], [13], [14], [15]. In particular Tenomodulin (TNMD) and Mohawk homeobox (MKX) have been suggested to mediate tendon cell differentiation, while Scleraxis (SCX) is indispensable for the initiation of tendon development. Mature tendon cells express both TNMD and SCX [11], and SCX expression has been shown to be dependent upon transforming growth factor-β (TGF-β) signaling. SCX is furthermore known to activate TNMD expression [16]. While not studied on MKX, both SCX and TNMD are linked to the mechanical and spatial extracellular environment [17], [18], suggesting an association with the mechanical environment and markers of the tendon phenotype.

Cell mechanotransduction determines multiple cell functions such as proliferation [19], differentiation [20], and wound healing [21]. To transmit signals from and to the matrix, cell-adherence to the extracellular matrix (ECM) is crucial and depends upon ligand binding by specific receptors. By this, cells can respond to the chemical, topographic and mechanical environment of the matrix [22]. At the same time, cell-matrix adhesions allow cells to act on the ECM by the development of actin-myosin mediated contractile forces and transmission of intracellular forces via receptor coupling from the cell to the matrix [23], [24]. One of the best studied families of ECM receptors are the integrin receptors [25], however in tendons, integrin receptors have hardly been investigated.

In the literature, four collagen-binding receptors are described, i.e. integrin α_1_β_1_, α_2_β_1_, α_10_β_1_ and α_11_β_1_ of which α_2_β_1_ and α_11_β_1_ primarily bind to collagen type I, the predominant collagen type in tendon [26]. Besides collagen, fibronectin constitutes the tendon ECM and is an important ligand to which integrin α_5_β_1_ binds. In tendon, the function and dynamics of cell-matrix receptors during collagen fibrillogenesis and in response to changes in the mechanical environment has not been investigated so far. In the event of injuries with full, partial and also micro-tears, the major mechanical stimulus in tendons is lost, or severely disturbed. Interestingly, in animal models, tendons have been shown to contract when tension is released [27], which was supported by a study on lax rat tail tendons that are actively contracted through an α-smooth muscle actin-mediated mechanism [28]. It is therefore likely that tendon cells interact and actively contract the ECM and thereby remodel the matrix after unloading. To what extent cell matrix adhesions are involved in the remodeling phase and in what way the release of tension affects the gene expression profile of human tendon cells is poorly described. It is furthermore not known whether initial cellular changes to alterations in the mechanical environment could activate a catabolic cascade. Previous studies have reported the induction of pro-inflammatory mediators and regulators of matrix degradation by fibroblasts in relaxed matrices [29], but this has never been investigated in response to de-tensioning of human tendon.

Thus, the purpose of this study was to investigate the response of human tendon cells to release of tension with regard to integrin receptor dynamics, the expression of matrix proteins and molecules associated with tendon cell differentiation. Furthermore, inflammatory mediators were studied in response to the lack of tensile loading. We applied an *in vitro* model in which tendon-like tissue is engineered with human tendon cells [30], [31]. We hypothesized that de-tensioning would lead to a decrease in integrin α_2_β_1_, α_11_β_1_ and α_5_β_1_, and that it would cause a decline in the expression of matrix components. We further hypothesized that the lack of the tensile stimulus would lead to a downregulation of mechanosensitive tendon cell markers and an upregulation of pro-inflammatory mediators. Lastly, we raised the hypothesis that a supplementation of TGF-β1 would stimulate the expression of collagen receptors and matrix proteins in both culture conditions, i.e. independent of the degree of mechanical tension in the tendon. The stimulating effect of TGF-β1 in lax tissue would thus reflect a fibrotic response of the tendon cells when the mechanical stimulus is diminished or absent.

## Materials and Methods

### Cell culture

Tendon fibroblasts were isolated from human semitendinosus and gracilis tendon as described previously [30]. Eight patients (18–32 years old) undergoing reconstruction surgery for anterior cruciate ligament rupture gave written informed consent and the experiments with human tissue were approved by the local ethical committee (den nationale videnskabsetiske komité, ref. H-3-2010-070). The investigation has been conducted according to the Declaration of Helsinki. After the tissue was harvested, it was immediately transported to cell culture laboratory. Under aseptic conditions, the tissue was minced in pieces of ∼1 cm^2^ and digested overnight in DMEM/F12 Glutamax supplemented with 0.1% collagenase type II (Worthington) and 20% fetal bovine serum (FBS) (Gibco, Invitrogen). Following repeated washes in growth medium (DMEM/F12 Glutamax, 10% FBS), cells were seeded into culture flasks, growth medium added and cultured until the next passage. Growth medium consisted of DMEM/F12 with 10% FBS. Cells in the 3rd to 5th passage were used for experiments.

### Fibrin scaffold preparation

Each well of a six-well plate was coated with ∼1.5 ml SYLGARD (Dow-Chemicals) and allowed to set at 55°C for 48 h. For the construction of fixed-length linear tendon-constructs, two short silk sutures (0.8 cm, Ethicon) were pinned onto the coated plates with minutiens insect pins (0.1 mm diameter) (Fine Science Tools GmbH) with a distance of 1.5 cm in between sutures. The plates were sterilized by immersion in 70% ethanol for 45 min. Human tendon fibroblasts were resuspended in DMEM/F12 Glutamax containing 4.0 mg/ml human fibrinogen and 0.5 units/ml of human thrombin (Sigma-Aldrich) to a final concentration of 2.5×10^5^ tendon cells in 600 µl construct-specific medium consisting of DMEM/F12 Glutamax with 10% FBS and supplemented with 0.2 mM L-ascorbic acid 2-phosphate and 0.05 mM L-proline (Sigma-Aldrich). From each cell line, a part of the cell suspension was saved to analyze the gene expression pattern under 2D and compare it to 3D conditions (see also under “Quantitative Real-Time RT-PCR”). 10 µg/ml aprotinin was added to the suspension and 814 µl of cell suspension with fibrinogen, thrombin and aprotinin was rapidly spread over the complete surface of the coated wells. The cell embedded fibrin gels were allowed to set for 60 min at 37°C, and cultured until the matrix was fully contracted, i.e. formed a continuous line between the two sutures, typically after 12–14 days. Every other day, construct-specific medium was replaced.

### 3D Tendon-constructs cultured under de-tensioned condition

**Figure 1:**
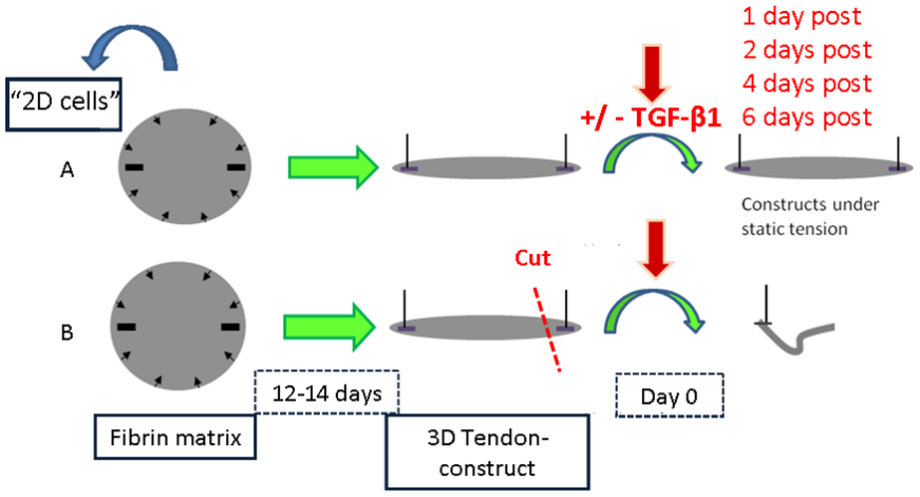
Overview of study design. Tendon cells on monolayer (“2D cells”) were harvested prior to seeding tendon cells into fibrin matrix to engineer human tendon-constructs. A) Culture condition under static tension, TGF-β1 supplementation to half of the samples at day 0 defined as the first time point at which a continuous linear matrix between the anchor points was formed; B) Tendon-constructs were cut to release tension at day 0, TGF-β1 supplementation to half of the samples.

To study the effect of tension-release, some of the 3D tendon-constructs were cut at one end. This was done at the first time point at which a continuous tendon-like tissue was formed between the anchors (figure 1). De-tensioned and tensioned tendon-constructs were cultured and harvested after six days (n =  5 (5 donor cell lines)), (figure 1). In order to investigate a potential time effect, the experimental setup was repeated and in this data set, sampling was done one, two, four and six days after de-tensioning (n =  3, (3 donor cell lines) for each time point). For all the different construct types, construct-specific medium was exchanged every other day.

### Supplementation with recombinant TGF-β1

At the first time point, at which the 3D tendon-constructs formed a continuous tendon-like tissue between the anchors, and some of the tendon-constructs were de-tensioned, the different constructs were divided into two groups (figure 1). In one group, 25 ng/ml TGF-β1 (recombinant human TGF-β1, Millipore) was added to the tendon-constructs, both to constructs under linear tension and to de-tensioned constructs. The construct-specific medium was changed every other day and fresh TGF-β1 was added each time.

### Quantitative Real-Time RT-PCR

**Figure 2:**
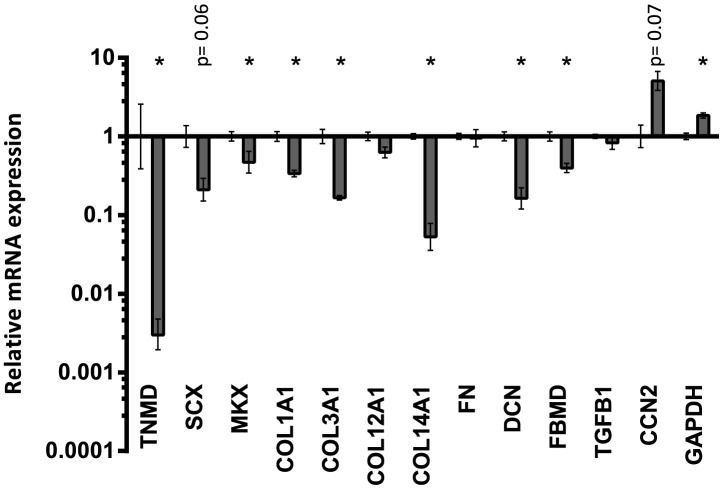
Comparison between 2D monolayer and 3D tendon-constructs under static tension. The 3D tendon-construct is set as baseline, and mRNA expression of tendon cells grown on 2D monolayer is shown relative to the 3D tendon construct. Significant changes are indicated by *. Data presented on a logarithmic y scale baseline as geometric means ± SEM with 3D tendon-construct as baseline (n = 5).

**Figure 3:**
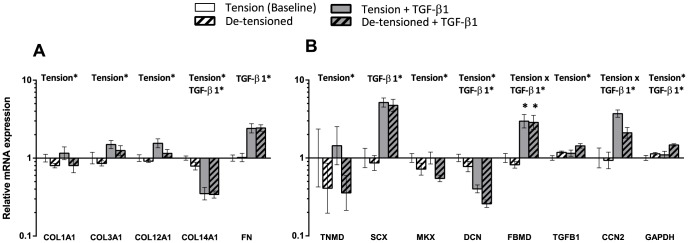
Summary of findings after de-tensioning of 3D tendon-constructs and TGF-β1 supplementation. The 3D tendon-construct under static tension without TGF-β1 is set as baseline, all other conditions are shown relative to the tensioned 3D tendon-construct (n = 5). A) Analysis of genes encoding for different matrix proteins: Collagen type I (COL1A1), collagen type III (COL3A1), collagen type XII (COL12A1) collagen type XIV (COL14A1) and fibronectin. B) Analysis of tendon phenotypic markers and tendon-related genes: tenomodulin (TNMD), scleraxis (SCX), Mohawk homeobox (MKX), fibromodulin (FBMD), decorin and GAPDH. Data presented on a logarithmic y scale as geometric means ± SEM with 3D tendon construct as baseline (n = 5). Significant 2-way ANOVA (tension*TGF-β1) main effects written above the graphs. For FBMD, * indicates significant effect of TGF-β1 for the individual groups in the post hoc analysis.

**Figure 4:**
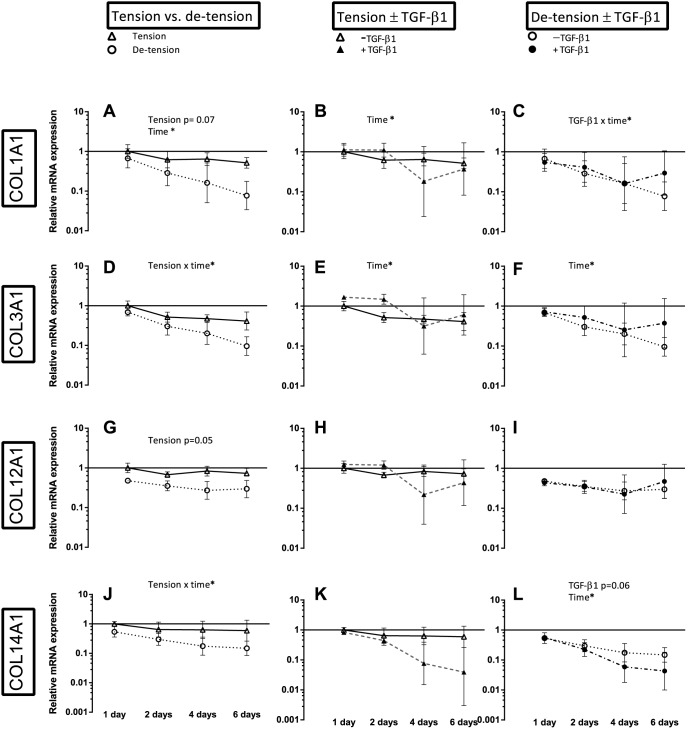
Overview over the effect of de-tensioning and TGF-β1 supplementation on mRNA expression of collagen genes. Left panel (A, D, G, J): Effect of de-tensioning, mid panel (B, E, H, K): Effect of TGF-β1 on tensioned 3D tendon-constructs, right panel (C, F, I, L): Effect of TGF-β1 on de-tensioned 3D tendon-constructs. The data are presented on a logarithmic y scale as geometric means ± SD (n = 3) where Tension Day 1 is baseline. Significant 2-way RM ANOVA (tension*time) or (TGF-β1*time) main effects written above the graphs. Open triangles: 3D tendon-construct under tension, without TGF-β1 supplementation; open circles: 3D tendon-construct de-tensioned, without TGF-β1 supplementation; filled triangles: 3D tendon-construct under tension, with TGF-β1 supplementation; filled circles: 3D tendon-construct de-tensioned, with TGF-β1 supplementation.

**Figure 5:**
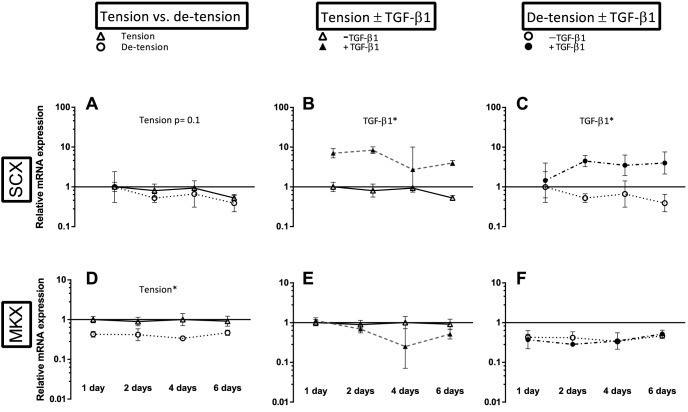
Overview over the effect of de-tensioning and TGF-β1 supplementation on mRNA expression of phenotypic markers of tendon lineage. Left panel (A, D): Effect of de-tensioning, mid panel (B, E): Effect of TGF-β1 on tensioned 3D tendon-constructs, right panel (C, F): Effect of TGF-β1 on de-tensioned 3D tendon-constructs. The data are presented on a logarithmic y scale as geometric means ± SD (n = 3) where Tension Day 1 is baseline. Significant 2-way RM ANOVA (tension*time) or (TGF-β1*time) main effects written above the graphs. Open triangles: 3D tendon-construct under tension, without TGF-β1 supplementation; open circles: 3D tendon-construct de-tensioned, without TGF-β1 supplementation; filled triangles: 3D tendon-construct under tension, with TGF-β1 supplementation; filled circles: 3D tendon-construct de-tensioned, with TGF-β1 supplementation.

**Figure 6:**
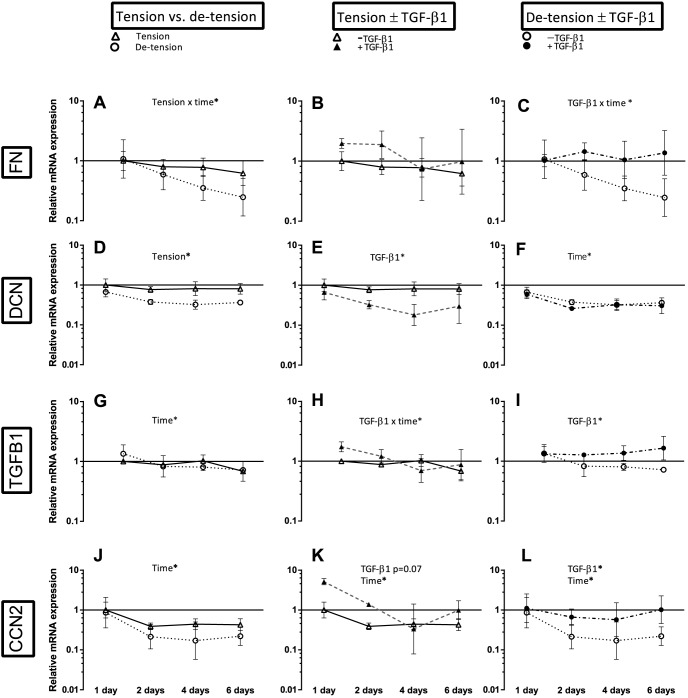
Overview over the effect of de-tensioning and TGF-β1 supplementation on mRNA expression of tendon matrix genes and growth factors. Left panel (A, D, G, J): Effect of de-tensioning, mid panel (B, E, H, K): Effect of TGF-β1 on tensioned 3D tendon-constructs, right panel (C, F, I, L): Effect of TGF-β1 on de-tensioned 3D tendon-constructs. The data are presented on a logarithmic y scale as geometric means ± SD (n = 3) where Tension Day 1 is baseline. Significant 2-way RM ANOVA (tension*time) or (TGF-β1*time) main effects written above the graphs. Open triangles: 3D tendon-construct under tension, without TGF-β1 supplementation; open circles: 3D tendon-construct de-tensioned, without TGF-β1 supplementation; filled triangles: 3D tendon-construct under tension, with TGF-β1 supplementation; filled circles: 3D tendon-construct de-tensioned, with TGF-β1 supplementation.

**Figure 7:**
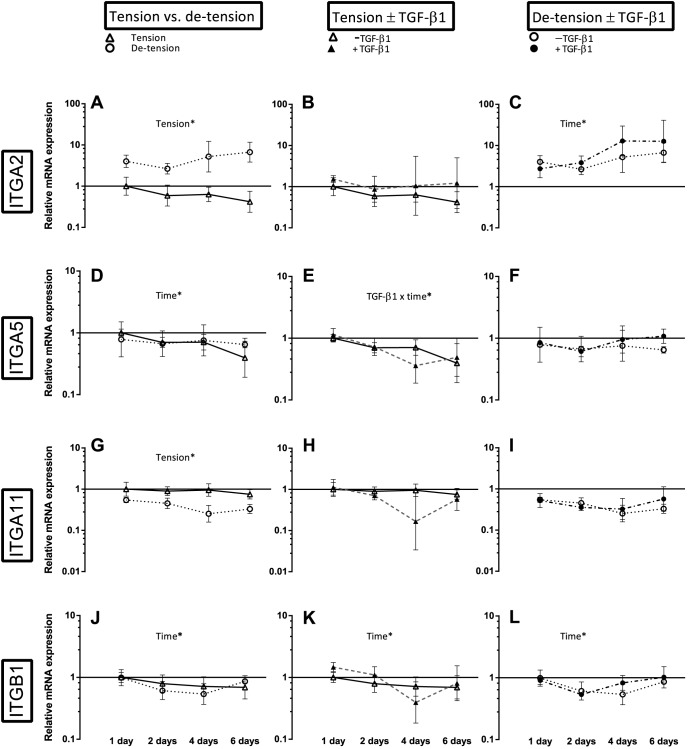
Overview over the effect of de-tensioning and TGF-β1 supplementation on mRNA expression of different integrin subunits. Left panel (A, D, G, J): Effect of de-tensioning, mid panel (B, E, H, K): Effect of TGF-β1 on tensioned 3D tendon-constructs, right panel (C, F, I, L): Effect of TGF-β1 on de-tensioned 3D tendon-constructs. The data are presented on a logarithmic y scale as geometric means ± SD (n = 3) where Tension Day 1 is baseline. Significant 2-way RM ANOVA (tension*time) or (TGF-β1*time) main effects written above the graphs. Integrin α_2_ (ITGA2); Integrin α_5_ (ITGA5); Integrin α_11_ (ITGA11); Integrin β_1_ (ITGB1). Open triangles: 3D tendon-construct under tension, without TGF-β1 supplementation; open circles: 3D tendon-construct de-tensioned, without TGF-β1 supplementation; filled triangles: 3D tendon-construct under tension, with TGF-β1 supplementation; filled circles: 3D tendon-construct de-tensioned, with TGF-β1 supplementation.

**Figure 8:**
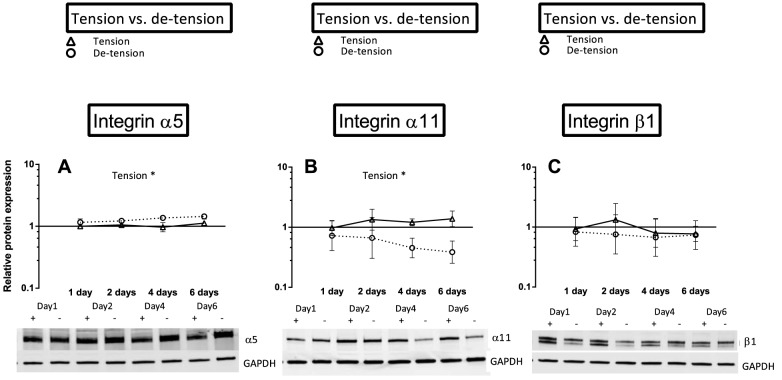
Overview over the effect of de-tensioning on protein expression of different integrin subunits. A) Effect of de-tensioning on integrin sub-unit α5, B) Effect of de-tensioning on integrin sub-unit α11, C) Effect of de-tensioning on integrin sub-unit β1. The data are presented on a logarithmic y scale as geometric means ± SD (n = 3) where Tension Day 1 is baseline. Significant 2-way RM ANOVA (tension*time) main effects written above the graphs. Open triangles: 3D tendon-construct under tension, without TGF-β1 supplementation; open circles: 3D tendon-construct de-tensioned, without TGF-β1 supplementation. Below the graphs, representative western blots are shown (+/- tension).

**Figure 9:**
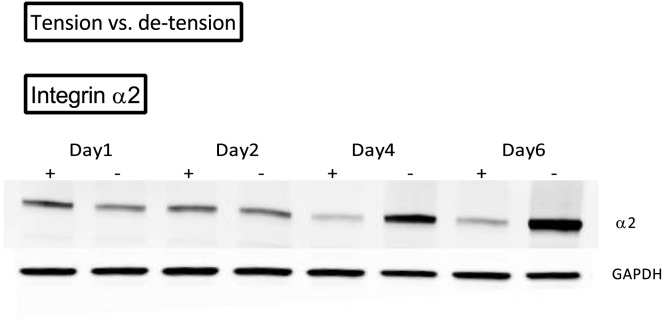
Overview over the effect of de-tensioning on protein expression of integrin subunit α_2_. The figure shows the western blot of one 3D tendon-construct over four time points shown(+/- tension).

**Figure 10:**
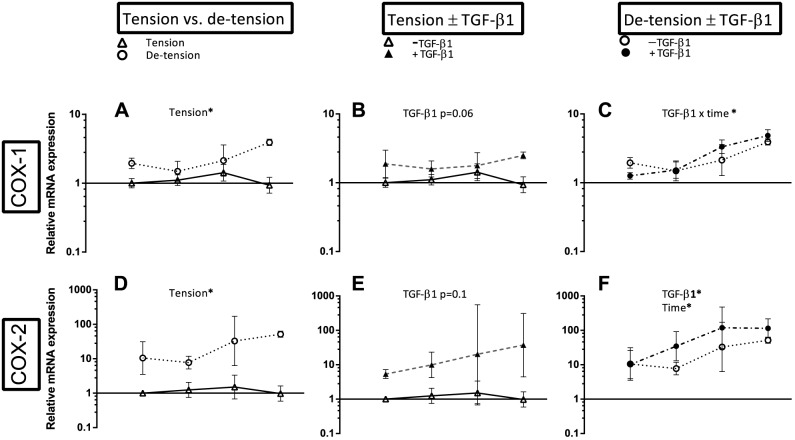
Overview over the effect of de-tensioning and TGF-β1 supplementation on mRNA expression of pro-inflammatory mediators. Left panel (A, D): Effect of de-tensioning, mid panel (B, E): Effect of TGF-β1 on tensioned 3D tendon-constructs, right panel (C, F): Effect of TGF-β1 on de-tensioned 3D tendon-constructs. The data are presented on a logarithmic y scale as geometric means ± SD (n = 3) where Tension Day 1 is baseline. Significant 2-way RM ANOVA (tension*time) or (TGF-β1*time) main effects written above the graphs. Open triangles: 3D tendon-construct under tension, without TGF-β1 supplementation; open circles: 3D tendon-construct de-tensioned, without TGF-β1 supplementation; filled triangles: 3D tendon-construct under tension, with TGF-β1 supplementation; filled circles: 3D tendon-construct de-tensioned, with TGF-β1 supplementation.

The amount of mRNA for target genes was measured with quantitative real-time reverse transcriptase (RT) PCR. An overview over targets and primers sequences is provided in table 1. Before embedding into fibrin gels, part of the tendon cells were transferred to TriReagent (Molecular Research Center, Cincinnati, OH, USA) to analyze gene expression before cells were exposed to different mechanical and chemical stimuli. The tendon-constructs were harvested, transferred to TriReagent and homogenized in 1 ml of TriReagent (Molecular Research Center, Cincinnati, OH) containing five stainless steel balls (BioSpec Products, Bartlesville, OK), and five silicon-carbide sharp particles (BioSpec Products), by shaking in a FastPrep-24 instrument (MP Biomedicals) at speed level 4 for 15 seconds. This was repeated three times with ice cooling between each shaking step. Following homogenization, bromo-chloropropane (Molecular Research Centre) was added to tendon cells and tendon-constructs in TriReagent, to separate the samples into an aqueous and an organic phase. Glycogen was added to the cells and constructs to improve RNA precipitation. Following isolation of the aqueous phase, RNA was precipitated using isopropanol, washed in ethanol and dissolved in RNAse-free water. RNA concentrations were determined using RiboGreen assay (Molecular Probes, Eugene, OR, USA). Synthesis of complementary DNA (cDNA) was performed using the Sensiscript reverse transcriptase (Qiagen, Hilden, Germany) on 30 ng of tendon cell RNA in a total of 20 µl. For each target mRNA, 0.25 µl cDNA was amplified in 25 µl Quantitect SYBR Green Master Mix (Qiagen) with specific primers (100 nM each, Table 1) on a real-time PCR machine (MX3005P, Stratagene, La Jolla, CA, USA). The thermal profile was 95°C, 10 min → (95°C, 15 s → 58°C, 30 s → 63°C, 90 s) × 50 → 95°C, 60 s → 55°C, 30 s → 95°C, 60 s. Signal intensity was acquired at the 63°C step and the Ct values were related to a 7-point standard curve made with known concentrations of DNA oligos (Ultramer™ oligos, Integrated DNA Technologies, Inc., Leuven, Belgium) with a DNA sequence corresponding to the sequence of the expected PCR product. Based on these standard curves and accounting for the PCR efficiency, the relative difference between unknown samples was determined. The specificity of the PCR products was confirmed by comparing the melt curves for the unknown samples with melt curves of the DNA oligos after amplification (the 55°C to 95°C step). The large ribosomal protein P0 (RPLP0) mRNA was chosen as internal control for normalization, and to test RPLP0 mRNA stability, another common “housekeeping” mRNA, Glyceraldehyde 3-phosphate dehydrogenase (GAPDH) was measured. The GAPDH/RPLP0 ratio was slightly higher in 2D versus 3D (figure 2) and also with release of tension and addition of TGF-β1 (figure 3B), indicating either an increase in GAPDH or a decrease in RPLP0. However, the changes were small compared to the changes in the targets of interest (figure 2–10) and RPLP0 was accepted for normalization. The normalized mRNA data were expressed as relative difference from the 3D tendon-constructs without TGF-β1 supplementation.

**Table 1:** Overview over primers used in the study.

mRNA	Sense	Antisense
**RPLP0**	GGAAACTCTGCATTCTCGCTTCCT	CCAGGACTCGTTTGTACCCGTTG
**GAPDH**	GCATTGCCCTCAACGACCACT	CCATGAGGTCCACCACCCTGT
**COL1A1**	GGCAACAGCCGCTTCACCTAC	GCGGGAGGACTTGGTGGTTTT
**COL3A1**	CACGGAAACACTGGTGGACAGATT	ATGCCAGCTGCACATCAAGGAC
**COL12A1**	CCCAGGTCCTCCTGGATACTGTGA	GCAGCACTGGCGACTTAGAAAATGT
**COL14A1**	AGCATGGGACCGCAAGGC	GACGCGCCACTGATCTCACC
**SCX**	CAGCCCAAACAGATCTGCACCTT	CTGTCTTTCTGTCGCGGTCCTT
**MKX**	CATCGTCATCAGAACTGAAGGCA	TCTGTTAGCTGCGCTTTCACCC
**TNMD**	GAAGCGGAAATGGCACTGATGA	TGAAGACCCACGAAGTAGATGCCA
**FN**	TTTGCTCCTGCACATGCTTT	TAGTGCCTTCGGGACTGGGTTC
**FBMD**	CAGTCAACACCAACCTGGAGAACC	TGCAGAAGCTGCTGATGGAGAA
**DCN**	GGTGGGCTGGCAGAGCATAAGT	TGTCCAGGTGGGCAGAAGTCA
**ITGA2**	TGGAATCCTTTTGCTGTTAGCTCTGG	CAGCTACTGAGCTCTGTGGTCTCATC
**ITGA5**	CTGTGGGGTGGCCTTCGGTTT	CGTCGCTTTGCGAGTTGTTGAG
**ITGA11**	CTCAGACGGTAGCATTGAGTGTGTG	GCTGAACTCAAAATCAAGACGGAAAG
**ITGB1**	GGATATTACTCAGATCCAACCACAGCA	TCAATGGGATAGTCTTCAGCTCTCTTG
**TGFB1**	GAGGTCACCCGCGTGCTAATG	CACGGGTTCAGGTACCGCTTCT
**IL1B**	TCCAGGGACAGGATATGGAGCA	AGGCCCAAGGCCACAGGTATTT
**COX-1**	GGTTTGGCATGAAACCCTACACCT	CCTCCAACTCTGCTGCCATCT
**COX-2**	AACTGCGCCTTTTCAAGGATGG	TGCTCAGGGACTTGAGGAGGGT
**IL6**	GAGGCACTGGCAGAAAACAACC	CCTCAAACTCCAAAAGACCAGTGATG
**MMP-9**	AGCGAGGTGGACCGGATGTT	AGAAGCGGTCCTGGCAGAAATAG
**MMP-13**	CCTGATGACGATGTACAAGGGA	TGGCATCAAGGGATAAGGAAGGG

Large ribosomal protein P0 (RPLP0); Glyceraldehyde 3-phosphate dehydrogenase (GAPDH); Collagen type I, α1 chain (COL1A1); Collagen type III, α1 chain (COL3A1); ; Collagen type XII, α1 chain (COL12A1); Collagen type XIV, α1 chain (COL14A1); Scleraxis (SCX); Mohawk homeobox (MKX); Tenomodulin (TNMD) ; Fibronectin (FN); Fibromodulin (FBMD); Decorin (DCN); Integrin α_2_ (ITGA2); Integrin α_5_ (ITGA5); Integrin α_11_ (ITGA11); Integrin β_1_ (ITGB1); Transforming growth factor-β1 (TGFB1), Interleukin-1β (IL1B); Cyclooxygenase 1 (COX-1); Cyclooxygenase 2 (COX-2). Interleukin-6 (IL6); Matrix metalloproteinase (MMP).

### Western blotting

Tendon cells cultured in monolayer were washed with Dulbeccós PBS, trypsinized and centrifuged at 600x g. After discarding supernatants, the resulting pellets were lysed in SDS-sample buffer and sonicated thereafter. Tendon-constructs were washed with Dulbeccós PBS, homogenized in SDS-sample buffer and sonicated thereafter. Protein concentration for monolayer cells and tissue was measured with the CB-X Protein Assay (G-Biosciences), and equal amount of protein subjected to a SDS-PAGE on Criterion XT Precast 4-12% Bis-Tris gels (Bio-rad). Electrophoresis was done at 200V for 60 minutes. Separated proteins were transferred onto PVDF membranes (GE Healthcare). Membranes were blocked for 30 min at room temperature (RT) with Odyssey blocking buffer (LI-COR) and incubated with the primary antibodies to integrin α_2_ (1:500, BD Biosciences 611016), integrin α_11_ (1:500, [32] ), integrin α_5_ (1:200, Santa-Cruz Sc10729), integrin β_1_ (1:500, Abcam ab52971) in Odyssey blocking buffer overnight at 4°C. Following washing in tris-buffered saline with 0.1% tween (TBS-T) three times for 5 min, GAPDH (1:4000, Invitrogen AM4300) was used as the second primary antibody and incubated for 120 min at RT. Upon washing in TBS-T three times for 5 min, membranes were further incubated with goat anti-mouse (Alexa Fluor 680, 1∶10,000, Invitrogen, 21057 or 31563) and goat anti-rabbit (Dylight 800, 1∶10,000, Thermo Scientific, 35571) for 60 min at RT. The integrin α_5_ antibody was added to the membrane after a stripping protocol using the Odyssey Stripping Buffer (LI-COR, 928-40032) for 30 min at RT, followed by blocking and antibody incubation as described above. Membranes were scanned in channel 700 and 800 using the LI-COR Odyssey device. Band intensities were quantified with the NIH-based image processing program Image J and normalized to the GAPDH band.

### Histology

Following two washes with PBS, 5 ml of sterile 10% formalin was added to each well in order to ensure fixation of the tendon-like tissue without disturbing the structure. The six-well plates containing formalin were transported to the pathology department. Once fixed with buffered 10% formalin, the pieces were dehydrated, embedded in paraffin wax in a longitudinal arrangement and cut into sections of 10 µm thickness. Finally, sections were stained with hematoxylin and eosin and photographed by white light.

### Electron microscopy

Culture medium was discarded and the tissue washed twice in Dulbeccós PBS, and the tendon-constructs were fixed by adding 5 ml of 2% Glutaraldehyde in 0.05 M phosphate buffer to each well. All samples were fixed for a minimum of 24 hours. After washing in 0.15 M phosphate buffer, the samples were post-fixed with 1% OsO_4_ in 0.12 M Sodium Cacodylate buffer for 120 minutes at RT. Following another washing in dH_2_O, the samples were stained en bloc with 1% aqueous uranyl for 16 hr at 4°C, dehydrated in a graded serious of ethanol, and embedded in Epon (Hexicon, Houston, TX). Ultrathin cross-sections were cut with a Reichert-Jung Ultracut E microtome using a diamond knife and were collected on one-hole copper grids with Formvar supporting membranes. Images were acquired in a Philips TM 100 transmission electron microscope, operated at an accelerating voltage of 80 kV, with a Megaview 2 camera, and processed with the iTEM AnalySIS software package (ResAlta Research Technologies, Golden, CO).

### Statistics

The level of statistical significance for all tests was p< 0.05. The mRNA and protein data were log transformed and analyzed using SigmaPlot 11.0 for Windows (Systat Software Inc., San Jose, CA, USA), graphical illustrations were performed with GraphPad Prism (GraphPad Software, San Diego, CA, USA). Comparison of the effect of the spatial environment (2D versus 3D) was tested by a paired Student´s t-test. For the first data set, the effect of tension and TGF-β1 supplementation was analyzed by two-way repeated measures. Where a significant TGF-β1*tension interaction was found, comparisons between time and tension, and tension and TGF-β1 within and across groups were performed using the Holm-Sidak method. When the post hoc test was not significant, main effects are written over the graphs, labeled as Tension* (main effect of tension vs. de-tensioning) and TGF-β1* (main effect of TGF-β1 supplementation). Data are normalized to the average level in 3D tendon-construct under tension, without TGF-β1 supplementation, and presented as geometric means ± backtransformed SE on a logarithmic y scale (n =  5) (figure 2 and 3). Significant ANOVA group effects are indicated by text in the figures.

For the second data set, the effect of time and tension and the effect of TGF-β1 supplementation and tension on mRNA level, or the different cell batches was analyzed by two-way repeated measures (RM) ANOVA. To simplify the statistical analysis, the effect of tension versus de-tension was analyzed separately from the effect of TGF-β1 supplementation. That is, two-way RM ANOVA was performed for time and tension (without TGF-β1 supplementation) and two-way RM ANOVA for time and TGF-β1 (for tension and de-tension separately). For the protein amount, the effect of time and tension or the different cell batches was analyzed by two-way RM ANOVA. Data are normalized to the average level in 3D tendon-construct under tension, without TGF-β1 supplementation, harvested at day 1, and presented as geometric means ± backtransformed SD on a logarithmic y scale (n =  3) (figure 4–10). Standard deviation was chosen due to low sample size (n =  3). Significant ANOVA group effects are indicated by text in the figures, for the effect of tension or TGF-β1, p-values indicating a trend (p ≤ 0.1) are included.

## Results

### Human tendon cells perform collagen neoformation in 3D tendon-constructs and reveal a different profile compared to tendon cells cultured on monolayer conditions

The mRNA expression pattern of human tendon cells cultured under tension in a fixed-length fibrin scaffold was compared to tendon cells in 2D cell culture. Figure 1 explains the experimental design: From the cell suspension taken for engineering of tendon constructs, a fraction was analyzed in order to compare the gene expression pattern of tendon cells only exposed to monolayer condition (2D) with engineered tendon-constructs (3D). When tendon cells were cultured on 2D monolayer (“2D cells”) and compared to cells cultured in the fixed-length 3D tendon-constructs, there was a dramatic downregulation in gene expression of the tendon-specific TNMD and the transcription factors MKX, as well as a trend towards a lower SCX mRNA expression (p =  0.06) in 2D cell culture. For collagen gene expression, COL1A1, (collagen type I, α1 chain) COL3A1 (collagen type III, α1 chain) and COL14A1 (collagen type XIV, α1 chain) were lower in expression on 2D, whereas there was no change for COL12A1 (collagen type XII, α1 chain). The mRNA encoding for the proteoglycans decorin (DCN) and fibromodulin (FBMD) were downregulated in cells cultured on 2D monolayer. GAPDH expression was higher in cells cultured on 2D monolayer (figure 2). For the other targets shown in figure 2, i.e. fibronectin (FN), TGF-β1 (TGFB1) and CCN2, there were no differences between cells grown on 2D monolayer and 3D tendon-constructs, although CCN2 revealed a tendency towards a higher expression on 2D monolayer (p =  0.07).

### The effect of de-tensioning and TGF-β1 supplementation for six days on human tendon cells

The effect of de-tensioning of the human 3D tendon-constructs was studied after a period of six days during which tension was released (n =  5) (figure 3A, B; table 2). The release of the static tension caused a spontaneous shortening of the tendon-constructs and further contraction over the six days. We found a decrease in the mRNA for COL1A1, COL3A1, COL12A1 and COL14A1 mRNA when tension was abrogated, but not for mRNA expression of the matrix protein FN. Importantly, there was no effect of TGF-β1 supplementation on the collagen genes COL1A1, COL3A1 and COL12A1 in the tensioned or the de-tensioned state, but a significant increase in mRNA expression for FN and a downregulation of COL14A1 mRNA (figure 3A). It was of interest to study if molecules associated with the tendon-specific phenotype were affected by de-tensioning of the tissue and we detected a decrease in TNMD and MKX mRNA expression when tension was lacking (p< 0.05), whereas SCX mRNA did not seem to change as a result of the de-tensioning (figure 3B). There was a clear effect of TGF-β1 supplementation on SCX expression, both in tensioned and de-tensioned tissue. The two proteoglycans included in the study showed an opposing effect of the treatment. DCN mRNA expression decreased when tension was lacking, and TGF-β1 supplementation caused a downregulation of DCN mRNA, whereas it had a stimulating effect on FBMD expression (tension x TGF-β1 interaction) (figure 3B).

**Table 2:** Summary of mRNA expression of tendon-related genes as a response to changes in the spatial/mechanical environment and TGF-β1 supplementation.

Target	Effect of 2D culture	Effect of de-tensioning	Effect of TGF-β1 supplementation
TNMD	↓	↓	↔
SCX	(↓)	↔	↑
MKX	↓	↓	↔
COL1A1	↓	↓	↔
COL3A1	↓	↓	↔
COL12A1	↔	↓	↔
COL14A1	↓	↓	↓
FN	↔	↔	↑
DCN	↓	↓	↓
FBMD	↓	Interaction tension x TGF-β1	Interaction tension x TGF-β1
TGFB1	↔	↑	↔
CCN2	(↑)	Interaction tension x TGF-β1	Interaction tension x TGF-β1
GAPDH	↑	↑	↑

Summary of figure 2 and 3: The effect of the intervention is compared to the gene expression of the respective target cultured for six days under static tension, the arrows (↑↓) indicate significant changes relative to the tensioned samples, or no statistical change (↔). Arrows in parenthesis indicate a tendency (p< 0.1). Tenomodulin (TNMD); Scleraxis (SCX); Mohawk homeobox (MKX); Collagen type I (COL1A1); collagen type III (COL3A1); collagen type XII (COL12A1); collagen type XIV (COL14A1); Fibronectin (FN); Decorin (DCN); Fibromodulin (FBMD); Transforming growth factor-β1 (TGFB1); Connective tissue growth factor (CCN2); Glyceraldehyde 3-phosphate dehydrogenase (GAPDH).

### The effect of de-tensioning with and without TGF-β1 supplementation on collagen gene expression over time

The observation of a negative impact on key molecules of tendon tissue and tendon tissue formation, when tension was released for six days (summarized in table 2), prompted us to study the effect of changes in the mechanical environment in a more detailed timeframe. Thus, we performed a new experiment with three different donor cell lines. We decided to include potential mechanosensitive targets and to test the hypothesis that the intervention would lead to a change in the inflammatory expression profile of the cells. As in the first experimental set, we studied the effect of TGF-β1 supplementation in the tensioned and de-tensioned state. Samples were collected after 1, 2, 4 and 6 days.

The release of the static tension caused a shortening of the tendon-constructs similar to the preceding experiments. Figure 4 shows the results for the fibrillar COL1A1 and COL3A1 and the FACITs (Fibril Associated Collagens with Interrupted Triple helices) COL12A1 and COL14A1, the figure summarizes the effect of de-tensioning (left panel), the effect of TGF-β1 supplementation in tensioned tissue (mid panel) and de-tensioned tissue (right panel). There was a lower mRNA expression of the fibrillar COL3A1 (tension x time interaction) and a trend towards a decrease in COL1A1 expression (p =  0.07) in response to the loss of tension (figure 4A, D). The fibril-associated COL14A1 revealed a decrease in mRNA expression when tension was lost (tension x time interaction), the fibril associated COL12A1 expression resembled that of COL14A1 as it tended to be lower when tension was lost (p =  0.05) (figure 4G, J). In addition, the mRNA expression of COL1A1, COL3A1, and COL14A1 declined over time when the cells were cultured in fixed-length 3D tendon-constructs under static tension (figure 4A, D, J). Just as it was seen in the first data set of experiments (figure 3A), there was no effect of TGF-β1 in the collagen genes encoding for COL1A1, COL3A1 and COL12A1 (figure 4B, E, H), when tension was present. In the de-tensioned tissue, COL1A1 had a higher mRNA expression when TGF-β1 was supplemented at the last time point (figure 4C). There was a tendency towards a lower expression of COL14A1 when TGF-β1 was supplemented in de-tensioned tissue, mirroring the findings from the first data set (figure 4L, figure 3B).

### The effect of de-tensioning with and without TGF-β1 supplementation on tendon markers

The samples collected over the four time points gave a similar pattern in gene expression of the tendon markers compared to the preceding experiment, i.e. MKX mRNA expression declined when tension was released, the transcription factor SCX showed the same trend (p =  0.1) (figure 5A, D). TNMD mRNA expression was below the detection level (i.e. less than one molecule per PCR reaction) in several samples in this set and thus did not allow for statistical comparison. In accordance to the findings from the first study, there was a clear effect of TGF-β1 supplementation on SCX expression both in tensioned and de-tensioned constructs (figure 5B and C).

### The effect of de-tensioning and TGF-β1 supplementation on matrix genes and growth factors

Besides different collagen types, we analyzed the tendon matrix genes FN, the proteoglycan DCN and the growth factors TGFB1 and CCN2 (figure 6). Both FN and DCN were negatively affected by the release of tension. As it was reported in the first data set (figure 3), TGF-β1 had a negative effect on DCN mRNA expression, but this effect was only present in the tensioned state (figure 6E). In the de-tensioned state, FN mRNA expression was stimulated by the pro-inflammatory/pro-fibrotic cytokine TGF-β1 (interaction TGF-β1 x time), as was TGFB1 itself and the growth factor CCN2 (figure 6C, I, L).

### The effect of de-tensioning with and without TGF-β1 supplementation on different integrin subunits

Engineered 3D tendon-constructs expressed mRNA encoding for collagen receptors ITGA2 (integrin subunit α_2_) and ITGA11 (integrin subunit α_11_) as well as the fibronectin receptor ITGA5 (integrin subunit α_5_) (figure 7), indicating that the tendon cells established cell adhesions through these receptors. As a result of de-tensioning of the tendon-constructs, the mRNA expression of the collagen-binding ITGA2 and ITGA11 showed a remarkable contrasting response to the release of tension. While the mRNA expression of ITGA2 level increased in the cells when tension was lost (p< 0.05), ITGA11 expression was decreased (p< 0.05), (figure 7A, D). It is important to note that the change in expression of both integrin subtypes occurred promptly, as ITGA2 mRNA was upregulated and ITGA11 mRNA downregulated already at the first time point. The mRNA expression of the fibronectin receptor ITGA5 did not change after de-tensioning of the tissue (figure 7G) and also the β_1_-subunit (ITGB1) mRNA expression remained unchanged as a result of de-tensioning of the 3D tendon-constructs (figure 7J). To confirm these changes at the protein level, we performed Western blot analysis of 3D tendon-constructs (figure 8 and 9). Our data showed that integrin subunit α_2_, α_11_ and β_1_ had similar profiles as their respective mRNAs with a significant difference between groups for integrin subunit α_11_ and no change for the β_1_-subunit (figure 8B, C). Interestingly, for the fibronectin-binding α_5_ subunit, we found a higher protein content after de-tensioning of the tissue (p< 0.05), (figure 8A), indicating a post-transcriptional mechanism being operative. The Western blot of integrin α_2_ revealed a higher protein amount of this integrin subunit when tension as released, confirming the mRNA data (figure 9). There was no effect of TGF-β1 supplementation on mRNA expression of the ITGA2 and ITGA11, but a drop in ITGA5 when TGF-β1 was present (TGF-β1 x time interaction) (figure 7E).

### The absence of tensile strain induces pro-inflammatory mediators

Cyclooxygenase-1 and 2 (COX-1 and COX-2) mRNA expression demonstrated both a significantly higher expression when tension was lacking (p< 0.05), (figure 10A, D), in particular COX-2 revealed a dramatic and rapid rise in the de-tensioned tendon-constructs (figure 10D). When the de-tensioned tissue was supplemented with TGF-β1, the increase in COX-2 mRNA was further pronounced (figure 10F). We observed a similar response for COX-1 (TGF-β1 x time interaction) (figure 10C). In the tensioned state, TGF-β1 tended to stimulate expression of both COX-1 and COX-2 (figure 10B, E). The effect of de-tensioning for mRNA expression of IL1B and IL6 resembled the pattern of COX-1 and COX-2 (figure S1A, D).

### Morphological characteristic in tendon-constructs under the different culture conditions

**Figure 11:**
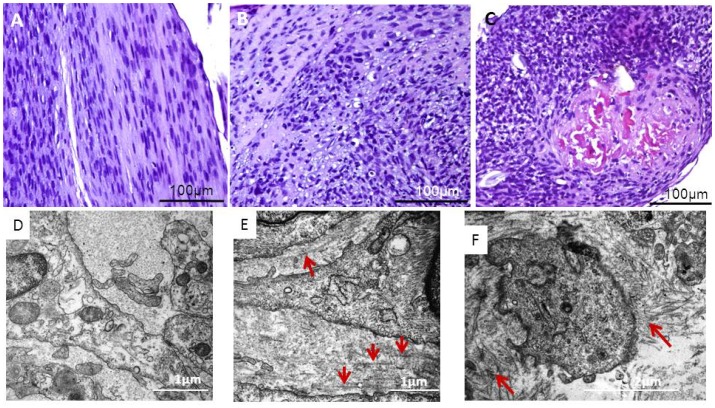
Visualizing of morphological changes to de-tensioning. A) H&E staining of 3D tendon-construct harvested 24 hours under static tension. (B) 3D tendon-construct harvested 24 hours after the release of tension, (C) 3D tendon-construct harvested six days after the release of tension. D) Electron micrograph of cross-sectioned 3D tendon-construct harvested after 48 hours under static tension. The extracellular space shows regularly organized collagen fibrils. (E) 3D tendon-construct harvested 48 hours after release of tension: Besides the presence of aligned collagen fibrils, disorganized collagen fibrils become apparent (red arrows). (F) 3D tendon-construct harvested 48 hours after release of tension: Extracellular space with disorganized collagen fibrils in close proximity to tendon cells (red arrows).

Three-dimensional tendon-constructs cultured under static tension demonstrated a linear parallel alignment of the matrix, and the cells had an elongated morphology, and were located in parallel with the matrix (figure 11A). After 24 hours in the absence of tensile strain, the matrix lost the parallel orientation and the tendon cells had a rounded morphology (figure 11B). A similar picture with even further dis-orientation was seen in the samples that were collected six days after de-tensioning (figure 11C). On electron micrographs, the change in the orientation of the collagen fibrils and the cellular arrangement became further apparent. In tendon-constructs under tension, collagen fibrils showed the characteristic regular alignment in between cells with collagen fibrils with a homogeneous size all running in parallel along the axis of strain (figure 11D). We could not detect any apparent changes in the morphology after 1 day of culture in de-tensioned matrices (data not shown), however, two days following the disruption of the tensile strain, the disorganization of collagen fibrils became apparent with longitudinally orientated collagen fibrils in between the organized network (figure 11E). Figure 11F reveals disarranged fibrils in close proximity of the tendon cell.

## Discussion

Tensile strain is a major mechanical stimulus in tendon tissue and the release of this stimulus leads to dramatic and rapid changes in human tendon cells. In this study, we investigated changes in human tendon cells between monolayer (2D) and tissue culture (3D culture), the effect of tissue de-tensioning and the result of TGF-β1 supplementation to engineered tendon tissue in tensioned and de-tensioned conditions. The focus was on tendon matrix components, cellular markers of the tendon cell phenotype and integrin receptors, in particular the collagen receptor integrin α_2_β_1_ and α_11_β_1_ as well as the fibronectin receptor integrin α_5_β_1_.

### Comparison of 2D monolayer with 3D engineered tendon constructs

Our results are based on tissue-engineered tendon-constructs. The main advantage of this tissue culture system is primarily found in two characteristics: The tendon cells are integrated in a three dimensional space and the collagen fibrils are synthesized and organized by the cells, which is in contrast to cell culture studies on monolayer or even 3D collagen gels, where cells interact with pre-formed collagen fibrils. In 2D culture, cells adhere to a rigid substrate and are geometrically constrained, in contrast to flexible 3D tissue cultures in which the cells can adopt a morphology, which resembles the *in vivo* structure [33]. As the 3D culture represents the natural spatial environment of tendon cells, we decided to show changes in gene expression when cells are cultured on monolayer relative to the 3D cell culture condition (figure 2). The comparison of tendon cells grown on 2D monolayer with cells embedded in 3D tendon constructs showed a dramatic down-regulation of the tendon differentiation marker TNMD and MKX, as well as several tendon related genes such as fibrillar and fibril-associated collagens (figure 2). The decrease in TNMD under 2D conditions has been reported earlier [11], [17], but importantly, we show in this report that human tendon cells have the capacity to re-express tendon differentiation markers in 3D tendon-constructs. The same was seen for important tendon matrix genes such as COL1A1 and COL3A1 and collagen fibril regulators COL12A1 and DCN (figure 2).

In contrast to a disorganized alignment of collagen fibrils under 2D conditions, tendon cells initiate collagen fibrillogenesis and organize a parallel fibrillar network in the 3D engineered tissue when cultured under static tension (figure 11A, D) [30], [31]. Our data suggest that the parallel arrangement involves integrin α_2_β_1_, integrin α_11_β_1_ and integrin α_5_β_1_ and formation of fibrillar adhesions (figure 7A, D, E, G, figure 8, 9) and further indicates a role of these receptors in cell-mediated organization of the ECM. Notably, integrin α_11_β_1_ has been identified in human embryonic mesenchymal cells in regions with highly organized collagen fibrils [34]. Moreover, Velling et al [35] showed that integrin α_2_β_1_ and α_11_β_1_ rescued a phenotype that was unable to deposit collagen fibrils. It seems as a plausible mechanism that tendon cells organize the parallelism of fibrils in the ECM through cellular forces and involvement of integrin receptors similar to what has previously been shown in fibronectin fibrillogenesis [36], [37]. While the presence and the distribution of the fibronectin receptor were described in tendon-constructs previously [30], its function, as well as the role of the collagen receptors, in tendon neoformation has remained elusive and need to be studied in detail.

### Response of tendon cells to de-tensioning

When tension was released, the two collagen receptors showed a contrasting response: There was a rapid decrease in integrin α_11_β_1_, yet an increase in integrin α_2_β_1_. Protein levels of the fibronectin receptor α_5_β_1_ protein levels increased then tension was abrogated, while the β_1_-subunit did not change after de-tensioning (figure 7 – 9). Together, these data suggest that the integrin receptors are mechanosensitive molecules, which are differentially regulated in tendon cells as a result of changes in the mechanical environment. Our findings are in accordance with previous reports on fibroblasts from other tissues, in which integrin α_2_β_1_ was increased during matrix remodeling and contraction [38]. It seems plausible that cytoskeletal forces are transferred through the α_2_β_1_ to the collagen fibrils in the matrix, thereby causing an active contraction of the tissue [38], [39], [40], [41], [42], [43]. The collagen receptor α_11_β_1_ however, requires mechanical tension as it is negatively regulated by tissue unloading in embryonic fibroblasts [39] and our data suggest that adult tendon cells show the same mechanosensitive behavior (figure 7G, 8A). The fibronectin receptor α_5_β_1_ has been linked to adhesion strength [44], [45], [46], but was reportedly not required for cell mediated collagen gel contraction [45], [47]. The contrasting outcome of the different subunits measured in our study is in accordance with findings by Roca-Cusachs et al [46] who reported different mechanical roles of integrins. The question how the changes in several integrin subunits affect downstream signaling pathways and targets is relevant and needs to be further elaborated in future studies.

Several studies have described an interplay between induction of the integrin α_2_β_1_ and inflammatory processes as well as the induction of MMPs [38], [48], [49], [50], [51]. Interestingly, Peters et al [52] revealed that integrin α_2_^−/−^ mice had a reduced joint inflammation in an arthritis model and a lower induction of MMP-3. We therefore chose to investigate the expression profile of pro-inflammatory molecules following de-tensioning. The pro-inflammatory genes encoding for COX-1 and COX-2 increased rapidly, in particular COX-2 showed a dramatic rise over time after the intervention (figure 10D), while we did not detect a change in MMP-9 and MMP-13 expression (figure S1). The increase in pro-inflammatory molecules is an intriguing finding, as it suggests that human tendon fibroblasts show signs of a transformation towards an inflammatory phenotype in a mechanically un-loaded condition. The plasticity of human tendon cells is further underlined by the robust changes in phenotypical markers of adult human tendon cells as a result of changes in the mechanical environment (figure 3, figure 5).

When the engineered tissue was de-tensioned, TNMD and MKX as well as several genes encoding for essential matrix proteins in tendon, decreased (figures 3, 4, and 6). It suggests de-differentiation of the tendon cells when a major mechanical stimulus is abrogated as both TNMD and MKX are associated with the differentiation into the tendon cell lineage. TNMD and MKX are expressed at a slightly later stage during tendon development than the transcription factor SCX, which is necessary for tenogenesis [15]. In the first data set (figure 3), we found no effect of the mechanical environment on SCX expression, indicating that the expression of this gene is not disturbed by alteration in the tensile load. In the time course experiment however, SCX expression showed a tendency to decrease when tension was absent (figure 5A). The lack of a clear response in SCX expression to changes in the mechanical environment is in contrast to previous reports, which showed SCX responsiveness to mechanical intervention [18], [53]. Maeda et al measured SCX expression in mice after transection of the Achilles tendon and reported a decrease in SCX expressing cells. Also the use of botulinum toxin A, and thereby a loss of skeletal muscle force, had a negative effect on SCX expression [53]. Notably, SCX expression in tendon cells was increasing again in the period when muscle force was recovering, suggesting SCX to be mechanosensitive. This is supported by Mendias et al [18], who found an increase in SCX when mice were subjected to treadmill running. The lack of a robust response of SCX in our data set could reflect the difference in responses between human tendon cells and tendon cells from rodents. Recent reports have suggested differences in the response of tendons in rats and humans *in vivo*. While rat tendon responded to a loading regime with an upregulation of several matrix genes [54], human tendons did not show the same reaction to loading, i.e. there was no change in any of the matrix genes included in this study [55]. It indicates human tendons to be more inert, i.e. less responsive to a given stimulus compared to animal tendons, but whether these findings can be transferred to in vitro studies remains speculative. Another factor might be that the time course over which we chose to study SCX expression should have been extended in order to detect significant differences.

### The effect of growth factor TGF-β1 on human tendon cells

During development, TGF-β1 signaling is a mechanism to maintain the tendon cell phenotype and is directly linked to SCX expression [16]. The lack of TGF-β1 was associated with a decrease in SCX expressing cells and transformation of these cells into another phenotype than tendon cells. We report a strong and rapid increase in SCX expression following the supplementation with TGF-β1, in both experimental setups (figure 3, figure 5), as well as in both the tensioned and de-tensioned state (figure 3, figure 5). In line with the response in de-tensioned tissue, Maeda et al [53] reported that SCX expression in tendon cells occurs independently of cytoskeletal tension. Somewhat surprisingly, our data do not support that increased SCX expression has a stimulating effect on other tendon cell markers such as TNMD and MKX (figure 3, figure 5), although a link between SCX and TNMD expression has been described previously [56]. Moreover, in our study we could not show a stimulatory role of increased SCX expression on the expression of COL1A1, which has previously been reported [13].

Tendon pathologies are often associated with the development of fibrotic tissue [57], [58], a process in which TGF-β is considered a key factor, in particular the TGF-β1 isoform [59], [60], [61], [62], [63], [64]. Following a tendon rupture, elevated levels of TGF-β1 have been documented [65], [66]. We tested in this study whether supplementation with TGF-β1 would cause a change in matrix gene expression in de-tensioned tissue, i.e. implying an overproduction of matrix components, and whether the effect of TGF-β1 supplementation would be different in the tensioned state. Our data show that TGF-β1 stimulates the production of FN mRNA (figure 3, figure 6C), and the growth factors TGFB1 and CCN2 in the de-tensioned tissue (figure 3, figure 6B, H, K). In addition, the rise in COL1A1 in de-tensioned tissue at the last time point following TGF-β1 supplementation indicates that the growth factor had a stimulating effect on collagen synthesis (figure 4C). Interestingly, TGF-β1 supplementation did not result in any changes of collagen expression in the tensioned tissue, suggesting a distinct regulation of the pro-fibrotic growth factor TGF-β1 in tissue in which the mechanical environment has been disturbed. It should be noted that the pro-inflammatory molecules COX-1 and COX-2 were even further induced in de-tensioned tissue when TGF-β1 was added (figure 10C, F). The integrin α_11_ subunit has recently been associated with myofibroblast differentiation, cell types that are tightly linked to tissue healing but also fibrosis [61]. In reports by Lu et al [67] and Carracedo et al [39] α_11_β_1_ levels were stimulated by TGF-β1, but we did not find the same pattern in our samples (figure 7I).

### Implications for conditions in vivo

Unloading of tissue can trigger a variety of pathological changes and we used the tendon and tendon cells to investigate the effect of de-tensioning on the cells. In tendon pathologies, the initial processes of tendon pathologies include focal changes in the matrix, also termed “microstructural damage patterns” [68]. These changes could cause collagen fibrils to become disintegrated, resulting in de-tensioned fibrils [68], [69], [70], and this in turn might cause changes in cell-matrix adhesions as well as a cell-mediated contraction of disintegrated collagen fibrils as a part of matrix remodeling [49]. We suggest that the de-tensioning *in vivo* alters tissue architecture, cell shape and orientation, and changes dynamics of integrin receptors as a part of remodeling of the tissue similar to what we demonstrated in this study. It is most likely that these initial processes activate pro-inflammatory mediators such as IL-1b, and members of the COX-pathway, thereby triggering a transformation towards an inflammatory phenotype. Our data underscore the ability of human tendon cells to undergo this transformation and indicate that the pro-inflammatory/pro-fibrotic growth factor TGF-β1 further impairs the cellular response in de-tensioned tissue. This study elucidates early changes of human tendon cells as a result of a change in the mechanical environment and gives indications of how tendon cells transform following a change in the mechanical environment. It appears logical that these changes occurring *in vivo* could hamper successful tissue regeneration and lead to the formation of fibrotic tissue.

## Conclusions

We report here that human tendon cells in a 3D tendon construct system perform collagen fibrillogenesis simultaneously with the production of collagen receptors integrin α_2_β_1_ and α_11_β_1_ and the fibronectin receptor α_5_β_1_. It seems likely that these receptors are involved in the organization of the collagen-rich ECM. When the tensile stimulus was removed, the integrin receptors showed a differentiated response, which likely reflects their different mechanical role in tissue contraction and remodeling. An increase in expression of pro-inflammatory molecules was detected and specific markers of tendon cell differentiation declined. This suggests substantial plasticity of the human tendon cells in response to changes of the mechanical environment. Further, it underlines the need for mechanical loading of tendon tissue to be present in order to maintain normal tendon structure and biochemical phenotype. Lastly, there was an effect of TGF-β1 supplementation on some tendon related genes. In the de-tensioned state, the changes observed after TGF-β1 supplementation might indicate an overproduction of matrix genes associated with fibrosis.

## Supporting Information

Figure S1 **Overview over the effect of de-tensioning and TGF-β1 supplementation on mRNA expression of pro-inflammatory mediators IL1B, IL6, matrix metalloproteinases MMP-9, MMP-13 and GAPDH.** Left panel (A, D, G, J, M): Effect of de-tensioning, mid panel (B, E, H, K, N): Effect of TGF-β1 on tensioned 3D tendon-constructs, right panel (C, F, I, L, O): Effect of TGF-β1 on de-tensioned 3D tendon-constructs. The data are presented on a logarithmic y scale as geometric means ± SD (n = 3) where Tension Day 1 is baseline. Significant 2-way RM ANOVA (tension*time) or (TGF-β1*time) main effects written above the graphs. Open triangles: 3D tendon-construct under tension, without TGF-β1 supplementation; open circles: 3D tendon-construct de-tensioned, without TGF-β1 supplementation; filled triangles: 3D tendon-construct under tension, with TGF-β1 supplementation; filled circles: 3D tendon-construct de-tensioned, with TGF-β1 supplementation.For IL-1b, 16% of the samples were below the detection level. For statistical calculation, these samples were set to the detection level (1 molecule in the PCR).
(EPS)
